# Myelin Oligodendrocyte Glycoprotein (MOG) Optic Neuritis: A Case Series

**DOI:** 10.7759/cureus.14452

**Published:** 2021-04-13

**Authors:** Masnon Nurul-Ain, Zuhratun Nazihah Khairul Kamal, Wan-Hazabbah Wan Hitam, Maimunah Abd Munaaim, Faizah Mohd Zaki

**Affiliations:** 1 Ophthalmology and Visual Science, Universiti Sains Malaysia School of Medical Sciences, Kota Bharu, MYS; 2 Ophthalmology, Hospital Kuala Lumpur, Kuala Lumpur, MYS; 3 Ophthalmology, Universiti Sains Islam Malaysia, Nilai, MYS; 4 Radiology / Pediatric Radiology, Universiti Kebangsaan Malaysia Medical Centre, Kuala Lumpur, MYS

**Keywords:** demyelinating diseases, optic neuritis, myelin-oligodendrocyte glycoprotein (mog)

## Abstract

Myelin oligodendrocyte glycoprotein (MOG) antibody disease has been recognised as a distinct demyelinating disorder. Optic neuritis has been reported as the most common presentation and manifestation of this spectrum disorder. This is a case series of three MOG optic neuritis patients. Patients involved are female with disease onset ranging between 7- and 37-year-old. Most of these patients experienced symptoms of profound reduced visual acuity with eye pain. All three patients had optic disc swelling upon first presentation and they experienced at least one episode of bilateral simultaneous optic neuritis. Only one patient had demonstrable optic nerve enhancement on magnetic resonance imaging (MRI). Disease was confirmed through positive MOG antibody. Patients typically responded well to intravenous methylprednisolone (IVMP) during acute attack of optic neuritis. However, one patient had suboptimal response to IVMP after multiple relapses. We noted multiple relapses of optic neuritis are common in MOG patients. MOG optic neuritis is a devastating, but treatable condition. Aggressive treatment during acute optic neuritis attack and relapse prevention may favour a good visual prognosis in MOG antibody disease.

## Introduction

Myelin oligodendrocyte glycoprotein optic neuritis (MOG-ON) has a distinct clinical characteristic [1]. Typical optic neuritis may affect 50% of multiple sclerosis (MS) patients with a known spontaneous natural recovery [2]. Atypical optic neuritis in MOG antibody disease (MOG-AD) and neuromyelitis optica spectrum disorder (NMOSD) are less likely to have a spontaneous resolution [3]. MOG-ON may offer a better visual outcome, compared to NMOSD [1,3]. Identification of optic neuritis characteristic in MS, MOG-AD and NMOSD is important as the visual prognosis and treatment approach varies. We present three cases of MOG-ON with emphasis on the demographic, clinical course, neuro-imaging findings and the visual outcomes of such presentation.

## Case presentation

Case 1

**Figure 1:**
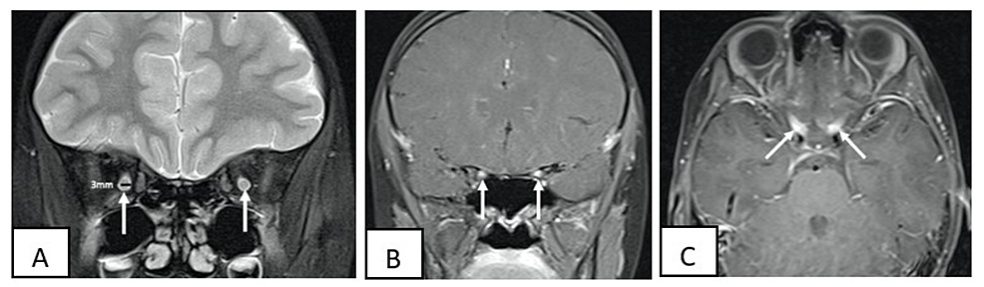
Coronal view showing both optic nerves are enlarged and measure 3 mm in diameter (normal for age is 2.37+0.44 mm) which shows abnormal T2 signal within (A) and enhancing on post-gadolinium (B). Axial view showing optic nerve enhancement on post-gadolinium especially at the intracanalicular portion, not affecting the intracranial portion (C).

A 7-year-old girl presented with reduced vision for three days with low-grade fever. There was pain on eye movement bilaterally with no symptoms of raised intracranial pressure. Visual acuity (VA) was 6/60 on the right eye (RE) and counting finger (CF) on the left eye (LE). Funduscopy showed a swollen and hyperaemic optic disc bilaterally. Systemic examination was unremarkable. MRI brain and orbit suggestive of bilateral optic neuritis with absence of associated brain parenchymal lesions (Figure 1). A diagnosis of bilateral optic neuritis was entertained. Intravenous methylprednisolone (IVMP) was administered for three days. Patient’s VA significantly improved. Serology analysis revealed positive anti-MOG antibody. The patient completed a tapering dose of oral prednisolone given over a period of six months. Latest follow-up after two years showed VA of 6/7.5 bilaterally with pink optic disc. No relapse of optic neuritis and patient remains stable with no other neurological issue.

Case 2

A 37-year-old lady presented with three episodes of optic neuritis. The first episode was bilateral, and subsequently the left eye was affected. During the attacks, optic neuritis was associated with severe headache and pain on eye movement. The patient also had low grade fever and generalised fatigue. VA was CF on the RE and 2/60 on the LE on the first episode of optic neuritis. Funduscopy revealed bilateral hyperaemic and swollen optic disc. The patient was given a course of IVMP and her VA improved to 6/9 bilaterally. Subsequently, during another two optic neuritis episodes, her VA profoundly decreased. However, patient’s VA improved after being given IVMP. MRI of the brain and orbit showed normal optic nerves with no associated brain parenchymal lesion. Serology analysis showed positive anti-MOG antibody. Corticosteroids treatment was tapered down slowly and the patient subsequently started on oral azathioprine. During the follow-up after six months of the latest relapse, bilateral VA was 6/9 and optic disc appears pink and normal. She remains stable with no other neurological complain.

Case 3

A 19-year-old girl had a total seven episodes of optic neuritis. She was initially diagnosed with acute disseminated encephalomyelitis (ADEM) at the age of 10. After one year, she developed LE optic neuritis with symptoms of reduced vision and pain on eye movement with low grade fever. VA was hand movement (HM) on the LE, and 6/6 on the RE. There was marked LE optic disc swelling and hyperaemia. She was given a course of IVMP and her LE VA returned to 6/6. She was in remission for four years before developing another six episodes of optic neuritis within a period of three years. The initial four episodes of right eye optic neuritis responded well to IVMP treatment. She was put on Azathioprine after the third optic neuritis attack. Subsequently, she had bilateral optic neuritis and another one episode of RE optic neuritis. In those two episodes of optic neuritis, VA remains poor despite being given IVMP. MRI brain and orbit were normal, while anti-MOG antibody was noted to be positive. The patient was started on intravenous immunoglobulin (IVIG) and her VA dramatically improved. She was then put on maintenance oral Prednisolone and Azathioprine. She is currently in remission for two years. Her VA was 6/12 on the RE and 6/9 on the LE. Fundus examination showed pale optic disc bilaterally with cup disc ratio of 0.3. She remains stable with no other neurological complication.

Table 1 shows the summary of the three patients concerning age and sex, optic neuritis attacks, visual acuity, neurological associations and treatment given to the patients.

**Table 1:** Summary of patients with MOG optic neuritis MOG: Myelin oligodendrocyte glycoprotein; RE: Right eye; LE: Left eye; CF: Counting finger; IVMP: Intravenous methylprednisolone; ADEM: Acute disseminated encephalomyelitis; IVIG: Intravenous immunoglobulin.

Patient, age, sex	Number of optic neuritis attacks	Bilateral simultaneous optic neuritis	Worst visual acuity at presentation	Visual acuity at final visit	Neurological associations	Treatment
Case 1, 7 years, F	1	Episode 1	RE 6/60, LE CF	RE 6/7.5, LE 6/7.5	None	Acute stage: IVMP for 3 days. Maintenance: Prednisolone tapered over a period of 6 months
Case 2, 37 years, F	3	Episode 1	RE CF, LE 2/60	RE 6/9, LE 6/9	None	Acute stage: IVMP for 3 days. Maintenance: Prednisolone 10mg daily, Azathioprine 100mg daily
Case 3, 19 years, F	7	Episode 6	RE CF, LE PL	RE 6/12, LE 6/9	ADEM	Acute stage: IVMP for 3 to 5 days, IVIG for 5 days given during episode 6 and 7. Maintenance: Prednisolone 10mg daily, Azathioprine 100mg daily

## Discussion

Incidence of MS and NMOSD has been widely reported world-wide [4-6]. However, little is known about the incidence of MOG-AD. A Dutch study reported the mean occurrence was 0.16/100,000 people, with higher seropositivity in children than in adults [7]. MOG optic neuritis (MOG-ON) was found to be 10% among the non-infectious optic neuritis cases in Japan and it stands for 13% of recurrent optic neuritis [8-9]. Recognition of this disease is important as patients are commonly presented with severe optic neuritis with frequent relapse which require different therapeutic approach. Patients in our series are all young females with the disease onset occurring between seven to 37 years old. This disease typically affects younger patients than NMOSD with median age of onset in the early to mid-thirties and more balanced female to male ratio [10-12].

MOG-AD is caused by damage of myelin oligodendrocyte glycoprotein, a membrane protein expressed on oligodendrocyte cell surfaces and on the outermost surface of myelin sheaths [1]. All of our patients had prodromal symptoms. Preceding infectious prodrome has been postulated to trigger the formation of MOG antibody. However, the role of infectious agents in triggering MOG-IgG production is still unclear. MOG antibody appears to mediate directly or activate the complement system, leading to central nervous system (CNS) inflammation and optic nerve demyelination [12-13].

Pain on eye movement and optic disc swelling on the first presentation are the striking features of MOG-ON in our patients. This corresponds to the previous report highlighting these two features as typical findings of MOG-ON [11-12]. Bilateral simultaneous optic neuritis attack which was previously reported to occur in 51% of MOG-ON patients [12], experienced by all of our patients. Optic neuritis has been reported to be the most common first clinical manifestation in MOG-AD. It is typically severe, but final good visual outcome after treatment, consistent with our findings. One of our patients had ADEM, while the other two did not show any other neurological manifestations. Although MOG-AD patients may develop myelitis, encephalitis and seizures, it has been reported that 30% of MOG-AD patients with recurrent optic neuritis may not show any other neurological symptoms [11]. Two patients in our series had multiple relapses. One patient developed relapse only after four years, which highlights that the occurrence of relapse might increase with longer follow-up. Relapses were previously reported to occur in 93% of patients with disease duration at or more than eight years [12].

In MOG-ON, optic nerve enhancement in MRI tends to be longitudinally extensive with perineural enhancement, usually sparing the chiasm and optic tract [1,11-12]. Only one of our patients demonstrated this finding. Negative findings of the other two patients were probably due to a delay of MRI appointment. The MOG antibody serology during the acute optic neuritis episodes was found to be positive in all of our patients. This highlights the importance of MOG antibody test as a diagnostic marker during the acute episodes, with high sensitivity and specificity [14].

The main principle of acute treatment in MOG-ON is to eliminate the systemic MOG antibodies [15]. Prompt initiation of treatment is also crucial in order to have a positive outcome [3]. Treatment usually consists of IVMP with the dose of 20-30 mg/kg/day for children, maximum 1-2 g for 3-5 days for adults [15]. However, administration of IVMP may not be as effective if patient has multiple relapses [1]. IVIG (total of 2 g/kg) over two or five days, or plasma exchange (PLEX) with five to seven exchanges on alternative days are the alternatives of therapy for those with suboptimal response to IVMP [15]. Disease modifying drugs (DMD) such as azathioprine, mycophenolate and rituximab need to be considered for those with relapse where it has helped to reduce the relapse rate in MOG-AD [1,11-12].

Poor VA was reported to be 16% (defined as VA of 6/36 or worse) to 36% (VA worse than 0.5) in at least one eye from previous studies of MOG-AD [10,16]. This highlights that aggressive management is the key to retain good functional vision and good quality of life in MOG-AD patients.

## Conclusions

MOG-ON affects young patients in our series, with high incidence of relapses. IVMP is generally effective in acute attacks, but multiple relapses may lead to treatment resistant and other treatment modalities such as IVIG or PLEX may be required. Long-term follow-up is important for MOG-ON patients, with prompt and adequate treatment as the key to attain a good outcome.
